# Institutionalizing grant-funded interventions: a multiple case study examining long-term investments in science, technology, engineering, mathematics, and medicine (STEMM)

**DOI:** 10.1186/s40594-026-00620-3

**Published:** 2026-06-08

**Authors:** Krystle P. Cobian, Naomi A. Stephen, Hector Ramos, Ana L. Romero, Sylvia Hurtado, Denise Ortiz

**Affiliations:** 1 David Geffen School of Medicine, University of California, Los Angeles, Los Angeles, USA; 2 Center for Developing Leadership in Science, University of California, Los Angeles, Los Angeles, USA; 3 School of Education and Information Studies, University of California, Los Angeles, Los Angeles, USA; 4 Diana Natalicio Institute for Hispanic Student Success, The University of Texas at El Paso, El Paso, USA

**Keywords:** Institutionalization, Program sustainability, STEMM training, Higher education, Student-centered programs, Grant-funded programs, STEMM interventions, STEMM education, Biomedical workforce

## Abstract

**Background, context, and purpose:**

Although funding agencies make large investments and encourage program adoption, little is known about the process of institutionalizing grant-funded efforts in higher education. This multiple case study examined how grant-funded awardees of a biomedical training grant worked toward institutionalizing program activities. Cases included 10 diverse postsecondary institutions (368 study participants) across the United States charged with implementing a federal grant aimed at comprehensive and multi-level approaches to engage and retain students from diverse backgrounds in biomedical research.

**Findings:**

Employing prior frameworks on institutionalization in higher education, we examined how embedded campus agents appealed to two determinants of institutionalization: compatibility and mutualism. We identified institutional resources as an additional determinant. Compatibility is most commonly sought after as early as the grant proposal stage, while agents typically appealed to mutualism in the later stage of implementation. Institutional resources are sought throughout all stages of the grant. Within each determinant, we describe strategies and tools that embedded agents use to facilitate long-term institutionalization of grant-funded efforts. We also describe common challenges sites faced as they worked to obtain compatibility, mutualism, and resources.

**Conclusions:**

Leaders of postsecondary science training initiatives who aim to institutionalize efforts can utilize a variety of strategies to enhance compatibility, mutually shared benefits, and committed resources to continue, embed, and/or scale the original initiative.

**Supplementary Information:**

The online version contains supplementary material available at 10.1186/s40594-026-00620-3.

## Introduction

Despite efforts to promote participation in Science, Technology, Engineering, Mathematics, and Medicine (STEMM^1^) fields, racial and/or gender underrepresentation persists (Chen et al., 2022; Fry et al., 2021; Ginther et al., 2011), prompting calls to address the structural barriers that reproduce inequitable career pathways and outcomes in STEMM (Chen et al., 2022; Fry et al., 2021; Robinson, 2022). Early attempts at enhancing diversity of participation in STEMM focused on providing targeted interventions to retain students from underrepresented groups in science disciplines (Gibbs et al., 2022). While such programs have contributed to individuals’ career advancement, they have also been criticized for trying to fix student-level issues rather than addressing the institutional and structural conditions that impede students’ capacity to progress into a STEMM career field (Handelsman et al., 2022; McGee, 2020; Robinson, 2022). STEMM education advocates have called for both programs and evaluation approaches that address systemic and organizational change (Lopez et al., 2022; Robinson, 2022), contending that without systemic change, efforts to remedy racism, sexism, and other forms of exclusion in STEMM will be ineffective (Espinosa & Posselt, 2021).

In a shift away from student-focused intervention programs, recent efforts have emerged that aim to create and study systemic changes in postsecondary science training (Carlisle & Weaver, 2018) and Reinholz & Apkarian (2018) (American Association for the Advancement of Science [AAAS 2026]; Sloan Foundation, 2021). This latest round of grant funding opportunities encourages innovations aimed at transforming postsecondary institutions by employing multi-level approaches to change that involve students, faculty, administrators, and community partners. Such efforts to broaden participation in STEMM implicitly or explicitly aim for deeper, transformative change that goes beyond sustaining the grant-funded activities Weaver et al., 2015 (Kezar & Gehrke, 2015).

Often grant funders encourage awardees to consider sustaining programs to maintain activities after the grant-funding period (Cutler, 2002). Yet, larger efforts to transform higher education encourage going beyond sustaining activities and toward deepening institutional transformation (Holland, 2009; Sandmann & Weerts, 2008). While sustaining a grant-funded program involves continuing major activities beyond the funding period, institutionalization is distinct in that it involves incorporating grant-funded activities into the normal, ongoing activities of the campus (Bailey et al., 2003; Cobian & Ramos, 2021). With these definitions in mind, it follows that working toward institutionalization of a grant-funded initiative facilitates institutional transformation.

The literature on organizational change at postsecondary education institutions primarily focuses on theories and broad case studies, with limited examination of the end-stages of institutionalization (Levine, 1980). The lack of research on how to achieve institutionalization, coupled with the increasing encouragement to institutionalize grant-funded initiatives poses a challenge to the growing number of grant-funded STEMM training initiatives. Yet, understanding institutionalization is essential if the long-term goal of a grant-funded initiative is to have successful innovations become permanent parts of the grant-funded institution(s). With respect to efforts aimed at broadening participation in STEMM fields, understanding institutionalization processes can improve the longevity of future grant-funded innovations that support STEMM recruitment, retention, professional training, and ultimately advancements in STEMM research.

This study examines how a network of postsecondary institutions, all recipients of the Building Infrastructure Leading to Diversity (BUILD) federal grant funded by the National Institutes of Health (NIH), worked to institutionalize their program innovations and activities. Employing multiple case study design, we examined the complexity of institutionalization, shaped by the interplay between the grant recipients’ agency within their institutions and the broader structure of each institution. We focused on how the sites worked toward institutionalizing grant-funded activities to enhance STEMM program participation and persistence of individuals from underrepresented backgrounds in biomedical research careers. We examined the following research question: How and when did sites work toward institutionalizing grant-funded innovations? We identified key strategies and challenges as sites worked toward institutionalizing grant-funded activities. Based on evidence from the sites, we expand earlier models of the process of institutionalization to highlight the importance of institutional resources. This study provides a theoretical and practice-oriented contribution to the literature on STEMM education.

### Background and overview of the BUILD initiative

In 2014, the NIH launched the Diversity Program Consortium (DPC), an experimental set of awards to gather data that have the potential to shape and inform science career training programs nationwide. The DPC consists of the Building Infrastructure Leading to Diversity (BUILD) program, the National Research Mentoring Network (NRMN), and the Coordination and Evaluation Center (CEC) (Valantine et al., 2016) and additional awards were granted in the second phase. For this study, we focused on the BUILD initiative.

BUILD awards differ from other NIH-funded training grants: rather than student- or trainee-focused interventions, BUILD was designed to achieve impact at the student, faculty, and institutional levels (see BUILD RFA-RM-13-016, 2013 https://grants.nih.gov/grants/guide/rfa-files/RFA-RM-13-016.html). Institutional eligibility included having less than $7.5 million in total NIH research funding and at least 25% Pell Grant recipient enrollment. BUILD awardees implemented and assessed evidence-based practices in recruitment and retention of underrepresented students while simultaneously building infrastructure and supporting faculty professional development (Valantine et al., 2016). Student-focused interventions include scholar programs, student learning communities, and peer and faculty mentoring (Eagan et al., 2024; Gibbs et al., 2022; Ramos et al., 2023). Several implemented activities were designed to be responsive to students’ needs, identities, and experiences (Cobian, et al., 2024; Hurtado et al., 2017). Examples of faculty development activities include mentor training (White-Lewis et al., 2022), pedagogical training, grant-writing programs (Hiatt et al., 2022), and pilot grants to support faculty research (Bienen et al., 2018). Institutional development consisted of renovations to create active learning classrooms, labs, and programming spaces, and efforts to scale up or expand activities such as undergraduate research and enhanced STEMM curricula.

Each BUILD awardee proposed and implemented site-specific approaches intended to engage students from diverse backgrounds in biomedical research, develop faculty mentoring and research skills, and promote institutional research and training capacity (Gibbs et al., 2022; Hurtado et al., 2017). Sites had an opportunity to apply for a five-year award renewal after the first five-year funding period (Gibbs et al., 2022). Thus, while most activities were implemented during the first phase of the grant, a few new activities were introduced during the second phase. The funding amount was decreased during the second phase of funding, as sites were expected to work toward sustaining successful interventions. Overall, the BUILD projects were encouraged to yield tangible advances in institutional research capacity, faculty development, and student research training (RFA-RM-13-016, 2013 https://grants.nih.gov/grants/guide/rfa-files/RFA-RM-13-016.html).

### Institutionalization

Institutionalization is parallel to long-term goals of funding agencies and advocates within the ecosystem of STEMM capacity building. Both phases of the BUILD projects included a focus on sustaining interventions developed during the funding period (RFA-RM-13-016, 2013 https://grants.nih.gov/grants/guide/rfa-files/RFA-RM-13-016.html) and yielding “transformative long-term changes at the institutional level.” We define institutionalization as the “process and outcome of iterative shifts between policies, practices, and aspirational values aimed at embedding an innovation as a permanent part of the institution’s actual values and practices” (Cobian & Ramos, p. 4, 2021). For this study, a BUILD activity was considered moving toward institutionalization if there was evidence of the following: the activity was currently funded or supported by institutionally funded staff to continue the activity, became embedded or absorbed into another office or department, and/or if the activity was scaled to reach a larger proportion of the student or faculty populations. Activities that were going to be maintained by additional external funding and activities where BUILD participants were still obtaining commitments were categorized as moving toward sustainability with the potential for future institutionalization.

How institutionalization is achieved in grant-funded STEMM training initiatives and pilot programs remains an important question. Grant-funded STEMM training initiatives examined from the lens of a life cycle (Bailey et al., 2003; Elrod & Kezar, 2016; Perez, 2023) ideally end with dissemination (sharing what is learned) as a final step (Elrod & Kezar, 2016) and/or institutionalization (Curry, 1991, 1992; Perez, 2023). An earlier case study of the BUILD initiative examined BUILD sites during early years of implementation and found evidence of sites actively working toward institutionalization while still implementing programs (Cobian & Ramos, 2021). While the study identified strategies to institutionalize programs, these strategies were not linked to broader organizational dimensions that incorporate the dynamic interplay between institutional agents’ efforts, resources, and the institutional contextual conditions (Stephen et al., 2026).

Minority-serving institutions (MSIs) have become an area of focus for capacity-building efforts (National Academies of Sciences, Engineering, and Medicine [NASEM], 2019; Palmer et al., 2013) because of their history of fostering talent despite being historically underfunded and underresourced. For example, while Historically Black Colleges and Universities (HBCUs) account for only 3% of colleges and universities in the U.S., they consistently outpace all other colleges and universities in the number of STEM[M] baccalaureate graduates who go on to obtain doctorates in science and engineering fields (Fiegener & Proudfoot, 2013; Taylor et al., 2017; Toldson, 2018, 2019). In addition to HBCUs, other MSIs cultivate STEMM talent at the predoctoral level (Einaudi et al., 2022; NASEM, 2019; Taylor & Wynn, 2019; Toldson, 2019). Despite having fewer financial resources, MSIs have a higher proportion of STEMM graduates from underrepresented groups compared to traditionally white institutions (Toldson, 2019). While MSIs support the participation and persistence of individuals from underrepresented backgrounds in STEMM, there is significant interest yet little research on how additional investments, such as grant funds that support STEMM training, might convert from extramural temporary funding to intramural sustained funding (Everley & Smith, 1996).

### Framework for the institutionalization of STEMM training initiatives

Specific actions and processes that occur during a grant-funded initiative can be critical to identify if material resources and partnerships (e.g., institutional financial support, physical space allocations, staffing commitments, and structured collaborations) will continue past the grant-funded phase. Institutionalization is often conceptualized as being a more complex, long-term process and outcome compared to program sustainability (Cobian & Ramos, 2021). While program sustainability is encouraged by funding agencies and/or institutional leaders providing seed funding for an innovation (Everley & Smith, 1996), many initiatives rarely move beyond sustaining their program activities and toward institutionalization (Levine, 1980).

**Fig. 1:**
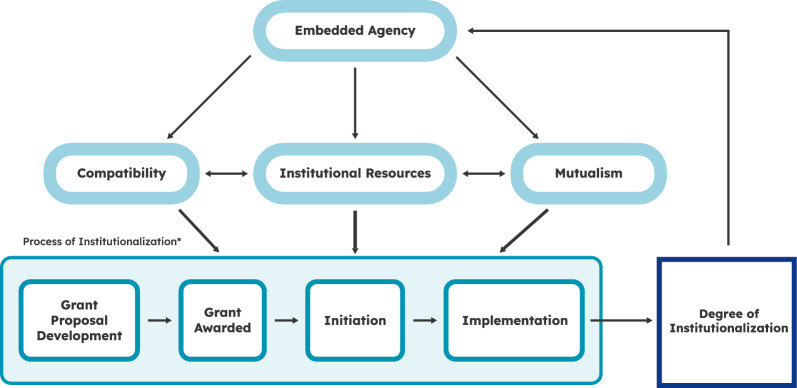
Model of Institutionalization of a Grant-Funded STEMM Intervention. Adapted from Ma & Cai (2021). Note: The process of institutionalization involves the iterative actions from embedded agents to increase compatibility, institutional resources, and mutualism during all stages of a grant, which contributes to the degree of institutionalization (extent to which grant-funded efforts are now embedded in the institution)

For this study, we adapted and conceptualized a model informed by prior models of institutionalization in higher education (Cai, 2017; Levine, 1980; Ma & Cai, 2021), scholarship on sustaining STEMM intervention programs (Okstad et al., 2023; Rincon & George-Jackson, 2016), and Resource Dependence Theory (Pfeffer & Salancik, 1978) (Fig. 1). We began with an early institutionalization model (Levine, 1980) and co-developed the findings of this study, pulling in additional literature and concepts through identified themes from the data. The final model depicts how sites moved beyond sustaining and worked toward institutionalizing grant-funded STEMM activities (Fig. 1).

After studying fourteen innovations in the experimental colleges at a state university in New York, Levine (1980) proposed two determinants of institutionalization of an innovation within a higher education context: “compatibility” (appropriateness of the innovation to the norms, values, and goals of the institution) and “profitability” (effectiveness of an innovation in satisfying adopter needs). Rather than “profitability" (Levine, 1980), we contend that “mutualism” is a more appropriate conception in that the focus is on mutually beneficial situations whereby both the innovating group and adopters perceive benefits that motivate their desire to maintain the innovation. Even in Levine’s original conception, profitability included subjective and noneconomic elements, such as “security, prestige, peer approval, growth, efficiency, and approval in the quality of life" (Levine, 1980, p. 18). Connecting literature about innovation in higher education to Levine’s research on institutionalization, Cai (2017) proposed a model for understanding the innovation-institutionalization process in higher education and included agency as a third factor that affects institutionalization. Agents include policymakers, managers, and academics who “change the existing institutional rules for facilitating innovation” (Cai, 2017, p. 606). Ma and Cai (2021) later adapted the concept of embedded agency (Seo & Creed, 2002) to account for the dynamic, complex set of linkages between institutional context and individual agency in generating institutional change. They placed embedded agency at the top of their model, where embedded agents influence the extent of compatibility and profitability, which in turn influences institutionalization. Strategies employed within each determinant of institutionalization consist of active and dynamic decisions and actions between individuals involved at each site. BUILD awardees as well as senior and central administrators at the sites can be thought of as embedded agents, or practitioners of praxis (Seo & Creed, 2002), which “points to a particular type of action, rooted in a collective consciousness that is conditioned but not determined by existing social arrangements (Seo & Creed, 2002, p. 240). With respect to STEM reform, researchers found that these “change agents” use several tactics to enact institutional change (Feola et al., 2023). While the makeup of the principal investigators (PIs) and BUILD core leaders at each BUILD site varied, faculty PIs and a few senior administrators (e.g., deans, assistant deans, assistant provosts) typically served as leaders for the BUILD program innovations. Their actions or inactions were the main drivers of appealing to determinants of institutionalization.

Adding to the prior models, we included institutional resources as a third determinant of institutionalization. In the process of transitioning from grant funding to “hard funding”, or institutional budgets (Everley & Smith, 1996), embedded agents work to establish legitimacy of their STEMM intervention programs (Rincon & George-Jackson, 2016) by acquiring resources to continue their work. Grant-funded teams use their agency to cultivate organizational change and navigate tensions between their efforts and structural challenges that limit their agency (Gomez et al., 2021; Okstad et al., 2023). While obtaining funding can contribute to a program gaining legitimacy on campus (Rincon & George-Jackson, 2016), obtaining administrative approval can also confer program legitimacy (Levine, 1980). Thus, linkages among resources, funding, staffing, and legitimacy become cyclical (George-Jackson & Rincon, 2012). These prior studies suggest that without internal or external stakeholders’ perceptions of the program as legitimate, securing resources over a sustained period can become difficult. Accordingly, a program’s potential for institutionalization is largely dependent on resources for its continuation (Pfeffer & Salancik, 1978).

Lastly, we propose that compatibility, mutualism, and institutional resources can be sought during the grant proposal development, grant award, initiation, and/or the implementation stage. Thus, we refer to efforts to increase compatibility, mutualism, and resources during any of these discrete markers of a grant as the process of institutionalization, meaning that institutionalization is both a process and an end goal. However, compatibility is most commonly sought after as early as the grant proposal stage, while agents typically appealed to mutualism in the later stage of implementation. Institutional resources are sought throughout all stages of the grant. We provide examples and expand on this work in the findings section.

## Methods

The CEC was tasked with serving as the central evaluation unit for the BUILD initiative. To evaluate the institutional aspects of the BUILD program, a research team within the CEC adopted a multiple case study approach (Stake, 2013). We describe case selection, additional details about the BUILD sites, our analytical decisions, positionality, trustworthiness, and limitations of the study.

### Case selection and site visits

The research team employed a multiple case study design (Stake, 2013). Case studies are useful for description of context-specific implementation of practices, including explanation, exploration, and replication. A multiple case study design generates findings from cluster comparisons that represent the larger phenomenon (Stake, 2013). Multiple case studies enabled us to understand how different contexts shaped institutionalization and how successes and challenges to institutionalize BUILD program elements converged and diverged across the 10 BUILD sites.

In 2022, research team members conducted online semi-structured interviews as well as 1- to 2-day site visits at each of the 10 BUILD awardee sites with a focus on understanding each site’s approach to institutionalization (Table 1). We obtained consent from all participants in the study prior to conducting interviews. The research team interviewed key senior administrators (e.g., president, provost, deans), principal investigators, BUILD program directors and staff, program participants (e.g., faculty, students, post-baccalaureate scholars), and program partners. Interviewees were generally asked about the impact of BUILD and the extent to which BUILD activities would remain a vital part of the campus after the grant ended (for protocol, see Supplementary Material 1). After site visits, the researchers conducted a debrief meeting with each BUILD team to share takeaways from the visit and clarify observations. These meetings served as a form of member checking (Lincoln & Guba, 1985; Onwuegbuzie & Leech, 2007). Interviews ranged from 45 to 90 min each via Zoom in spring and summer 2022, and in-person interviews and focus groups took place in fall 2022. Data consisted of transcripts of virtual and in-person interviews, observation notes, documents (e.g., photos, website summaries, news articles, reports), analytic memos, and narrative reports. Interviews and focus groups were recorded, transcribed, and uploaded to Dedoose software for analysis. In total, the research team conducted 238 interviews and 19 focus groups with a total of 368 participants. Different forms of evidence (photos, observations, etc.) confirmed assertions from interviews and focus groups.

**Table 1:** BUILD sites and institutional characteristics

Institution	Institutional type and minority serving institution status	Undergraduate enrollment	Pell grant recipients	Case study participants (n = 368)	
				Faculty, staff, and senior administrators:	Students & post- baccalaureate trainees:
Site A	Public HSI/AANAPISI	36,979	54%	28	7
Site B	Public HSI/AANAPISI	32,025	51%	25	6
Site C	Private HBCU	2,344	54%	29	18
Site D	Public HSI	20,004	57%	25	8
Site E	Public Emerging HSI	19,648	40%	31	7
Site F	Public HBCU	6,294	56%	17	8
Site G	Public AANAPISI	11,144	28%	18	5
Site H	Public HSI/AANAPISI	25,867	45%	33	12
Site I* Site K *(Research university partner)*	Private Catholic N/A Public N/A	(I): 11,144 (K): 16,728	(I): 29% (K): 45%	20	48
Site J	Public ANNH	5,432	21%	14	9

Institutional Characteristics based on 2020 data from the U.S. Department of Education. AANAPISI = Asian American Native American Pacific Islander-Serving Institution, ANNH = Alaska Native/Native Hawaiian-Serving Institution, HBCU = Historically Black College and University, HSI = Hispanic Serving Institution. Emerging HSI = Hispanic students make up between 15 and 24 percent of full-time undergraduates. Site E was recognized as an AANAPISI in 2023

^*^Sites I and K were a joint BUILD award, with Site I serving as the primary site

### Coding and cross-case analysis with analytic framework for institutionalization

**Fig. 2:**
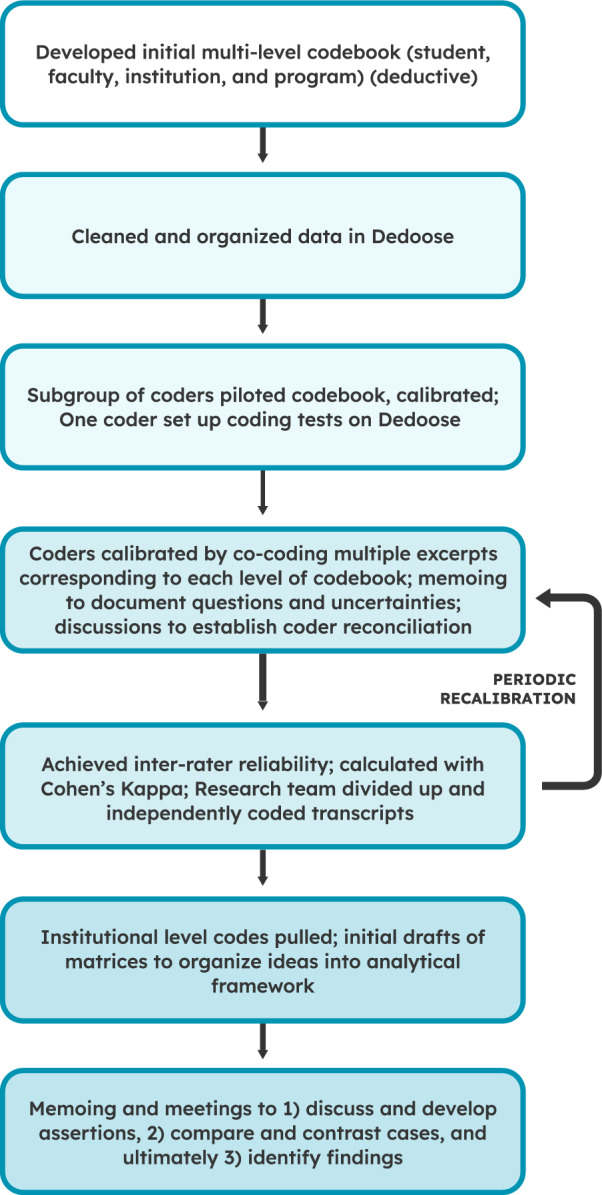
Coding and Analysis Process

Figure 2 shows the steps involved in our coding and analysis. First, we deductively developed a draft of the codebook to focus on institutionalization, sustainability, and program impact since these were overarching topics of interest for the broader case study (see Supplemental Material 2). Prior to conducting the site visits, we developed codes to account for institution-wide, program-specific, faculty-specific, and student-specific level codes and themes. This step enabled us to ground the codebook and interview protocols within STEMM education research and organizational frameworks. A subgroup of three coders piloted the codebook using an initial set of transcripts. One research team member set up four coding tests on Dedoose for each level of codes within the codebook. A broader group of five coders met regularly over the course of six months following the site visits to revise and recalibrate the codes and definitions to inductively capture unique BUILD processes informed by the interviews and focus groups (Hemmler et al., 2022). We used Dedoose to calculate interrater reliability using a Pooled Cohen’s Kappa coefficient (Cohen, 1960) for each coding test. Once the team coders engaged in a series of discussions to establish coder reconciliation, the team reached greater than 0.80 for tests at each level (Cole, 2024). The team then independently coded the 238 transcripts.

We co-developed the analytic framework for institutionalization with data analysis to arrive at our findings and the final analytic model for institutionalization. The framework for institutionalization (Cai, 2017; Levine, 1980; Ma & Cai, 2021) served as a theoretical guide in that it provided a map for qualitative exploration (Miles & Huberman, 1994; Saldana, 2015). We utilized analytic matrices, a tool that enables researchers to visualize the data, its frequency, and compare qualitative differences across sites and/or embedded cases (Miles & Huberman, 1994). To develop the matrices, we pulled and reviewed the coded excerpts on institutionalization, site debrief reports, case narrative reports, and analytic memos, re-coding and reorganizing the data into a matrix to identify how sites worked toward institutionalization. This allowed us to see similarities and differences within and across BUILD sites. Next, we organized the data into the key components of the analytic model to better understand how embedded agents appealed to compatibility and mutualism (named *profitability* in the Levine, 1980 model) by site. We moved between the organizational framework and the data reiteratively, developing additional questions, themes, and subthemes. This led to us to identify institutional resources as a third thematic determinant of institutionalization missing from the previous literature, and distinctions in the stages of the grant when embedded agents appealed to compatibility, mutualism, or worked to obtain resources. The co-authors presented initial assertions to the larger CEC team for feedback, questions, and additional context, which served as a form of peer review of research internal to teams to enhance the reflexivity and validity of the findings.

### Positionality

We position ourselves as education researchers who care deeply about STEMM equity. The co-authors were all members of the CEC, funded by NIH to evaluate BUILD, and involved in the overall evaluation of the BUILD program. While outsiders to STEMM departments, the co-authors have studied and written about organizational change in STEMM postsecondary contexts. All co-authors identify as Latine and/or multiracial, four as first-generation college students, and two earned bachelor’s degrees in a STEMM discipline. Collectively, our life experiences have influenced our interest in examining institutional change to foster STEMM equity and shaped our interpretation of BUILD data. Throughout the case study visits, debriefs, and analysis, we raised questions with peers about the extent to which our perceptions of site visits may have been biased by our individual experiences and social positioning. We provide more details in the Trustworthiness and Limitations sections regarding how we addressed interpretations within this study.

### Trustworthiness

After each site visit, the case study team created a debrief report highlighting emerging themes regarding institutionalization and program impact at the site. The site visit researchers met with each BUILD site’s program leaders within a month of conducting the in-person visit to discuss and clarify themes. This enabled an opportunity to offer insights and clarify observations made during the site visit. We then updated the debrief report and sent a final copy to each site so that each campus could use it to further their initiatives. Additionally, the case study team wrote case narrative reports for each site, which serve as a context-specific analytical tool to develop assertions that begin to answer the broader study questions for each site (Stake, 2013). Lastly, members of the site visit team kept jottings and memos in a secure, shared cloud storage location to begin to make sense of observations (Corbin, 2016) during the second phase of case study data collection.

To triangulate findings, we converged evidence from interviews, documents, observations, and follow-up consultations with participants to verify details. For example, if a participant shared that an activity was institutionalized, we searched the data for confirming or disconfirming perspectives from other participants at the site and for details of how the activity was institutionalized. If participants mentioned plans to institutionalize a BUILD activity, we followed up to find press releases, announcements, or other online information to confirm that the final steps of securing material resources and institutional commitments had occurred. As an additional method of ensuring trustworthiness, we searched for divergent cases to account for nuanced differences in the findings.

### Limitations

We provide limitations and clarifications about the scope of the study to ensure transparency and to place our findings and implications in context. First, a thorough analysis of the quality, fidelity, and participant outcomes of each BUILD site’s multiple BUILD-funded activities was beyond the current study’s scope due to our need to focus on identifying activities that would become institutionalized and analyzing the institutionalization process. Future research can connect BUILD outcomes such as biomedical graduation rates, persistence into biomedical graduate study, and measures of psychosocial outcomes and changes to campus climate with institutionalized BUILD activities. Next, most participants were interviewed once, potentially limiting our understanding of how the process of institutionalization shifted over time during BUILD implementation. However, we did interview some participants (mainly PIs and faculty heavily involved in BUILD implementation) virtually prior to site visits, and again 2–3 months later during the in-person site visit. This allowed us to gain additional insight about updates and changes to BUILD implementation and the campus context.

## Findings

We describe how and when sites worked toward three key determinants of institutionalization: compatibility, mutualism, and institutional resources. Within each determinant, we provide examples of how embedded agents at each site worked toward institutionalization by appealing to the three determinants. Throughout the findings, we summarize the overall patterns found from the sites, highlight an example from a site that best exemplified the theme or a case that went against the overall pattern. We also provide a high-level summary of the cross-case analysis findings (Table 2) and a table with the themes, subthemes, and strategies (Table 3).

**Table 2:** Cross-case analysis of findings

Site	Compatibility	Mutualism	Institutional resources
Site A	Aligned with mission, norms, and values of institution–focus on equity Challenge: several STEMM training programs on campus and leadership turnover made it difficult to obtain consistent leadership support	Demonstrated success of grant-developed innovations to support transfer student population– led to obtaining additional institutional and external grant-funded commitments	Obtained institutional commitments to establish an undergraduate research office
Site B	Developed coalitions with STEMM colleges and STEMM training programs on the foundation of shared goals	Convinced system-wide leadership to commit support due to win–win situation: grant-funded faculty training success supported system-wide aims to foster faculty teaching and mentorship skill development	Obtained institutional commitments from senior administrators to continue student-focused and faculty-focused training activities
Site C	Aligned with mission and norms of institution; emphasized reputation of STEMM disciplines on campus	Grant-funded tracking and evaluation efforts made it easy to demonstrate success of grant-funded activities Challenge: institutional type (small private) and budget posed challenge to maintaining successful post-baccalaureate training program	Obtained institutional commitments to continue academic support activities for students
Site D	Aligned with institution’s research mission and norms regarding supporting students’ potential	Rigorous evaluation of grant-funded curricular innovations showed success; convinced institution to maintain grant-developed courses	Obtained financial commitment from senior administrators to continue a scholarship program for STEMM undergraduates
Site E	Aligned with goals to mitigate barriers for students– focus on financial barriers	Convinced state legislature to commit additional funds due to win–win situation of grant activities also achieving state’s needs	Obtained financial support from college deans Obtained commitments to develop space inside a renovated building on campus to continue grant-funded activities
Site F	Aligned grant activities with strategic plan– focus on enhancing research and becoming R1	Demonstrated positive impact of STEMM training center activities developed by grant and convinced senior administration of positive results	Obtained financial and physical resources from institution’s senior administration, and an additional federal grant Context: former PI of grant was promoted to a senior administrative position during the middle of the grant
Site G	Aligned with institutional culture and reputation for STEMM training and education innovation	Fostered a win–win collaboration with faculty development office on campus, outcomes met needs of BUILD aims and faculty development office aims	PI leveraged STEMM departmental and college budgets to embed grant-developed activities and positions into college Continued collaboration with faculty development office to continuously provide training program to STEMM faculty
Site H	Aligned mission of grant with institutional mission, norms, and values– large focus on equity Challenge: grant project perceived by institution to be “niche” and only benefitting a small group of students	Fostered mutually beneficial partnership with research partner– consistent group of student trainees that matriculate into training programs at partner site	Challenge: institutional budget cuts Unable to obtain institutional financial commitments
Site I and Site K (Research university partner)	Aligned with goals to develop STEMM training in the local geographic region	Grant-funded faculty development activities also achieved aims of senior administrators’ goals to support new faculty hires	Site I: Obtained approval for curricular innovations and funding for an additional 1–2 years to support student-focused activities Site K: Obtained financial commitments from institution for undergraduate scholarships for STEMM students
Site J	Aligned with strategic plan focused on enhancing access to research experiences for students, strengthening indigenous programs, & achieving R1 status	Fostered a win–win collaboration with both institutional leadership and tribal partners– led to grant initiative evolving into a new master’s program	Obtained an additional federal grant to maintain some student-focused activities

**Table 3:** Themes and Subthemes for the Process of Institutionalization of the BUILD Initiative

Theme	Subthemes	Strategies
Compatibility: Degree to which the grant-funded innovation(s) are consistent with the norms, values, and goals of the organization	Partners and/or senior leadership external to grant team perceive alignment between institutional plan and grant aims to enhance participation in biomedical research Shared agreement on *incompatibility* with the way things are at the institution (e.g., desire from institution to shift toward becoming a research university) Challenge: shared mission to support STEMM training may exist, but there is misalignment of goals to achieve mission of how to equitably serve a diverse student population Challenge: compatibility exists, but difficult to obtain support amid other STEMM initiatives on campus	Develop Memorandums of Understanding (MOUs) Establish advisory boards for initiative(s) Build or leverage consistent communication channels with STEMM deans and/or other STEMM training programs on campus Obtain commitments from leaders and/or campus partners based on shared mission, goals, and values Find common ground and shared goals with resistant leaders and/or potential campus partners
Mutualism: Degree to which needs are perceived to be satisfied by outcomes of the grant-funded innovation(s)	Mutual benefits perceived and acknowledged by one or more offices or centers on campus Outcomes are perceived as effective by institutional leaders, partners, and/or collaborators Challenge: low capacity to generate evidence of mutually beneficial impact Challenge: communicating mutually beneficial impact to audiences	Rigorous evaluation of innovation(s) Participation tracking Create and/or leverage opportunities to communicate successes Obtain commitments based on win–win outcomes for both grant project and on-campus partner(s) and adapters
*New Determinant:* Institutional Resources: Degree to which institutional human, financial, structural, and infrastructural resources were available	Available physical spaces (e.g., offices, labs, lounge space) for continuing activities of grant-funded innovation(s) Financial resources from the institution Structural/staffing resources from the institution Note: when institutional resources were not available or accessible, the degree to which external resources were obtained (grant funds, fundraising, etc.) Challenge: resources are limited due to institutional circumstances	Obtain commitments for offices and/or centers on campus to absorb cost and/or labor of activity (e.g., faculty training, undergraduate research training workshops) Obtain financial commitments from institutional budgets to fund activity Hire grant-funded staff into permanent roles on campus with expectations to continue aims of original grant Commitments to physical space and/or financial support to establish new centers or offices on campus to continue aims of original grant
Embedded Agency: Collective action by a group of institutional challengers	Embedded agents include senior leaders that support grant-funded innovation(s) and individuals on the grant-funded team who work to challenge and change institution Leveraging knowledge and experience of grant-funded team to navigate political and bureaucratic challenges	Obtain a deep understanding of institutional norms, challenges, barriers, and strategic plan Take initiative to find opportunities for collaboration and funding Determine which institutional leaders (provosts, deans, directors, etc.) can be of support to grant-funded innovation(s) Involve senior leaders in the grant proposal development Persistence Creativity Develop and elevate leadership within the grant-funded team Advocate for various ways to communicate impact (e.g., research publications, websites, infographics, presentations, etc.)

### Compatibility: achieving alignment with institutional goals

As a key determinant of whether an innovation will become institutionalized or not, compatibility is the measure of the appropriateness of an innovation within the existing institutional boundaries (Levine, 1980). While it does not determine whether an innovation will work, it does indicate the degree to which the innovation is consistent with the norms, values, and goals of the institution. We found evidence of several sites employing strategies to increase compatibility by aligning BUILD goals with institutional goals. Efforts to enhance compatibility were most commonly employed during the early stages of a grant’s life cycle—the proposal writing stage, the awardee status stage, and initiation stage of implementation. We identify key subthemes that provide nuance on how and when site leaders appealed to compatibility and discuss common challenges affecting compatibility.

The most common way of increasing the compatibility of BUILD’s initiatives/goals with the institution was by building coalitions with others on campus. Often, this included deans of colleges in science and health-related disciplines, senior administrators, influential faculty members with strong social networks, and directors of offices and centers on campus that served students and faculty. Two sites (B & E) exemplified use of this strategy as they brought together leaders from several STEMM training programs on campus to streamline recruitment and selection into these programs and create shared programs that served all students, regardless of existing training program. Sites also had to coordinate shared priorities among STEMM colleges on campus. One participant at Site B reflected on the process:

*To be honest, at first there was a lot of infighting. The colleges didn't really trust each other; we had different resources; we had different priorities. But I think, through BUILD, we learned to work together and then we put the BUILD mission in front and now it's more of a collaborative.*

While this work required patience and communication, these sites illustrate that it is possible to find common ground among colleges and/or STEMM training programs and work together to pool resources to remain focused on implementing and maintaining STEMM training activities.

Support from senior administrators who were external to the grant team also contributed to compatibility. While three sites had the benefit of having senior administrators serving as PIs, other sites actively worked to ensure that senior administrators at their respective institutions perceived institutional or departmental strategic plans as being aligned with BUILD’s goals to build biomedical research training capacity. We saw several examples of BUILD leaders actively communicating how BUILD aims aligned with departmental, college-level, or institutional priorities. Yet, communication is merely the start; BUILD leaders also created structures to solidify action toward areas of alignment in values and aims, sometimes in creative ways. For example, a few sites utilized memoranda of understanding (MOUs) to obtain commitments to meet regularly with senior administrators to implement BUILD. Unique from the other sites using MOUs, BUILD team members at Site B explained that MOUs also mitigated personnel turnover challenges. Thus, if a senior administrator departed, the likelihood of continuing efforts was high. This was important because most campuses experience turnover in senior administrative leadership. Additionally, the provost’s office at Site I had a goal of improving the faculty search process. This aligned well with the BUILD site team’s innovation of allocating funds and efforts to recruit faculty search advocates into the college of science on campus. As a result of the shared goals between the provost’s office and the BUILD initiative, the institution was able to institutionalize faculty search advocates across campus. Furthermore, Site F’s BUILD team leveraged institutional norms around utilization of advisory boards. While most grant-funded efforts and higher education institutions have advisory boards, this practice was most pronounced at Site F and used intentionally as a strategy to create formal coalitions and lines of communication with community organizations, industry partners, and senior administrators. These varied approaches suggest that compatibility can be achieved through various strategies, whether or not senior administrators were involved as PIs or were external to a grant-funded initiative.

BUILD teams that worked to create structures and processes to increase communication and buy-in from senior administrators saw more success in obtaining compatibility compared to other BUILD sites. Embedded agency can be seen when examining the interplay between BUILD team leaders and senior administrators who were not part of the grant-funded team. For example, Site B’s senior administrators were highly aware of BUILD activities and were actively involved in regular meetings with BUILD leaders. Despite senior leadership changes over the course of the grant, there was continuity in communication and involvement. For example, a new provost was hired midway through the grant funding period and prioritized their involvement and recognition of BUILD’s goals. The incoming provost actively participated by attending BUILD meetings, provided suggestions and ideas to envision other ways to secure resources and maintain BUILD-initiated programs at the site. This was intentionally structured into the provost’s job description and ensured active engagement of senior administration. This kind of built-in awareness and influence in the job description of senior administration is critical in that it elevates the status of the grant-funded initiative and ensures it is on the radar of key senior leaders. At Site B, continuing activity expectations with the next provost hire was a strategic way to ensure both compatibility and regular discussion of institutional material resources for BUILD activities over time.

Shared *incompatibility* is also important for aligning goals. Interestingly, goals to shift an institution’s Carnegie classification status created high compatibility with BUILD efforts. For context, three sites (F, G, J) were in the process of achieving intensive research status during the NIH-funded BUILD period (R1, Carnegie Classification of Institutions). In 2014, at the start of the BUILD initiative, Site F was classified as R3 but acquired R2 status in 2018 with plans to progress toward R1 status. Thus, efforts to enhance diversity in biomedical research were well-aligned with not only the mission of the institution, but also with each site’s strategic plan to increase research and graduate degree production. A senior administrator who is actively involved in supporting Site F’s BUILD efforts shared how the progression toward R1 aligns well with the success of scaling BUILD efforts, and how the program has partially contributed to broad-scale institutionalization:

*I'm not saying it's all because of [BUILD], but partly because of [BUILD]. We were an R3 institution in 2014. We are now an R2 institution, and the president has made a goal that we will go toward an R1 institution. Before [BUILD], we didn't have anything called the Office of Undergraduate Research. Now the [senior administrator] has made a determination that we will have an Office of Undergraduate Research to help students…The [senior administrator] has made a determination that we will be given small awards of various sorts to faculty members to initiate research… because [site F] was a teaching institution.*

Senior administrators felt that pushing for increased research activity at their sites would afford them additional resources and opportunities. Thus, BUILD program efforts were met with buy-in from senior administrators at the BUILD sites that were aiming to increase their research productivity.

Sites that didn’t have this buy-in faced additional struggles in their efforts to appeal to compatibility. Given that several of the BUILD sites are MSIs with missions that historically aim to focus on teaching and supporting students within their local geographic communities, BUILD’s initiatives to enhance diverse participation in the biomedical research workforce may appear well-aligned with the awardee sites’ institutional missions. Upon closer examination, we found cases of perceived misalignment or incompatibility of goals. An appeal to mission and vision at institutions, particularly at MSIs which often proclaim their capacity to be student-centered, is not enough to ensure that STEMM training program activities are compatible with broader institutional goals. Despite a shared mission that prioritizes equity and access to opportunities for students, differing opinions of achieving these aims can contribute to low compatibility between the institution and BUILD-funded project. As a result, there is less support from institutional leaders and collaborators to institutionalize BUILD activities. For example, when BUILD was considered too “niche”, or was perceived as a program that allocated resources to a small number of individuals who might benefit, it did not garner support from key institutional agents with influence and/or resources to allocate toward institutionalization. The senior administrators at Site H, for example, communicated their emphasis on wide-reaching approaches to equity and perception of BUILD as too narrow. Relatedly, Site A felt the pressure to stand out as an exemplar among the several on-campus programs with similar goals of supporting equity, access to opportunities, and student success. One participant from Site A shared the following:

*This year we have a new [senior administrator]. So, it takes a long time to let administrators know about our program. And then when they come in, there's just like 40 programs… our [other senior administrator’s] answer literally was, "Yeah, there's a lot of people doing good work on campus”... it’s like, "You're just like everyone else." And I thought, "We are, but except for this is a unique opportunity... why are we not seeing it that way?"*

Even when an innovation aligns with the institution’s mission, vision, or goals listed in the institution’s strategic plan, an initiative may still encounter resistance rather than support that is crucial to institutionalizing BUILD innovations.

### Mutualism: fostering win–win situations with outcomes

Appealing to mutualism consists of fostering win–win situations whereby adopters also obtain benefits or have needs met, ultimately garnering their support to continue the innovation. While compatibility is focused on *shared goals,* regardless of outcomes of the innovation, mutualism is distinct in that the innovation’s *outcome(s)* are perceived as beneficial to the innovators and the adopters. The benefits of the grant-funded initiative are perceived and acknowledged by one or more offices or centers on campus, or perceived as effective by institutional leaders, senior administrators, and/or campus partners. If the outcomes are perceived as beneficial, then these key parties are more likely to support and/or scale up the activity to allow it to continue beyond the grant-funded period. Efforts to enhance mutualism were commonly employed during the implementation stage. BUILD teams appealed to mutualism by providing evidence of program impact and ensuring that program activities were mutually enhancing outcomes for other partners and centers on campus. Challenges included little capacity to generate evidence of mutually beneficial impact and communicating the mutually beneficial impact to audiences. We elaborate on the strategies and challenges in the next section.

Sites appealed to mutualism by providing evidence of the impact of BUILD activities on achieving aims shared by both the initiative and the institution at large. Most often, program evaluation results were the mechanism for demonstrating success. Two sites were particularly effective at employing this strategy. Site D invested in revamping the institution’s first-year science curriculum to include original research experiences. Evaluation of the curriculum changes showed increased student retention, convincing institutional leaders that this grant-funded innovation helped achieve the institutional goals to increase undergraduate research training and retention. Site C employed rigorous and systematic participation tracking of BUILD-funded tutoring and supplemental instruction in STEMM courses, which they connected to student outcomes that demonstrated an association between better student outcomes and participation in the grant-funded activities. In both cases, evidence from evaluation of the grant-funded innovation led to senior administrators perceiving the benefits of the innovation, which ultimately led to commitments to invest the resources necessary to maintain the structure and staffing required of the innovation’s associated activities.

In getting potential adapters to perceive an innovation as beneficial, one challenge we identified is having the capacity for obtaining the appropriate data, analysis, and insights that showed evidence of successful outcomes. While all sites were required to evaluate their programs, the number of program activities occurring at multiple levels (student, faculty, and institutional level) left sites with the challenge of having to determine where their evaluators should focus their efforts. Additionally, sites were challenged by dividing evaluation time between reporting data to the central evaluation center (the CEC) for the project and conducting their local evaluations. Lastly, outside circumstances can disrupt evaluation efforts. For example, an evaluator at Site B shared how COVID-19 challenged their progress toward measuring outcomes:

*We had this idea in 2019… that maybe something we can do is quantify the progress you're making every year and see are we on track? But then COVID hit. It’s like, how do you quantify? Everything changed. A lot had to be put on hold. And then how do you quantify that?*

Evaluation and implementation were impacted by COVID at all the sites, but this evaluator’s account highlights what several sites faced—the challenge of examining and measuring outcomes before sites could even determine when and where to share results of their intervention activities.

Next, obtaining satisfaction from as many individuals as possible supported mutualism. We found that faculty mentor training initiatives were more likely to be institutionalized at sites where not only centers, departments, or senior leaders perceived the activity as beneficial, but the users themselves also reported multiple benefits of participating in the activity. Site B, evaluation showed that faculty members reported improvements to their mentorship and teaching because of participating in the faculty mentor training. Similarly, at Site C, faculty members shared how their participation in mentor training was critical to their professional development and ultimately, tenure and promotion. One potential explanation for their success in achieving mutualism for adapters is that the BUILD team members at the two sites tailored faculty mentor training and experimented with differing formats to continuously improve its use for adopters. Another potential explanation for faculty at these two sites perceiving the training as beneficial is that teaching and mentoring are weighed more heavily in tenure and promotion compared to a research-intensive institution. Regardless of the rationale for adopter perceptions, it was clear that the degree to which participants perceived the grant-funded activity as beneficial helped garner more support to maintain the activity.

Several sites demonstrated success by providing public venues for students, staff, and faculty involved in BUILD program activities to share how the program impacted their lives. Each BUILD site had a communications coordinator or team that focused on outward-facing materials to communicate the successes of BUILD. At Site E, the investment of time and energy to communicate the BUILD story led to commitments from both institutional leaders and external donors with shared aims to support the success of students from underrepresented groups in STEMM.

While some sites were successful in communicating success and obtaining buy-in from showing mutually beneficial outcomes, other sites faced challenges in successfully communicating the mutually beneficial impact to multiple audiences or did not prioritize this dissemination work. For example, a senior administrator at a BUILD site expressed confusion about the BUILD team’s institutionalization plans:

*I don't know what they're doing to institutionalize… I've institutionalized several grants, and I think that what you generally need as a plan is to say, "Here's the things that we really want to continue funding in a sustainable way. How do we incrementally make those things happen? “Here's the outcomes of the things that we've done. This is why you should invest in it.” But again, those might be conversations that they're having, just not having them with me. I just don't know.*

This senior administrator suggested planning and taking the initiative to communicate outcomes and provide suggestions to sustain program activities. Upon going back to review the data to triangulate this account, we confirmed that other senior administrators at this site were also unable to reference specific BUILD institutionalization plans. Some sites were at times overwhelmed managing multiple priorities with implementation that communicating program outcomes often got set aside or delayed. Interestingly at this same site, the same senior administrator shared that BUILD was doing a good job of communicating their successes:

*BUILD definitely gets tons of press and tons of praise, and I think it is very appreciated. I definitely think that happens… They’re good at communicating their successes. I think they're well known. I just don't know how integrated they are.*

Here, the administrator expressed a lack of knowledge regarding how BUILD was working toward integrating into university operations. It is important to note that leadership turnover did hamper communication of goals and plans with senior leaders at some sites, which may have contributed to the disconnect between sharing outcomes yet not making progress on institutionalization. However, it seems that a key step is to not only ensure that the audience perceives the program outcome as mutually beneficial, but to also ensure that the audience is aware of the initiative’s intentions to sustain itself and convinced to support efforts to sustain or institutionalize the program.

Another approach was for the BUILD-funded activity to provide beneficial outcomes for several entities on campus. For example, Site J centered a paradigm in their BUILD program called One Health, a paradigm that integrates human, animal, and environmental health. The paradigm was an important innovation in that it attracted a greater number of students, including Indigenous students, to see themselves as scientists. BUILD team leaders began working during the early years of the grant to establish a master’s program based on the One Health paradigm. A BUILD leader at the site shared how the BUILD-funded weekly One Health seminars eventually became part of the master's program, and thus funded by the institution:

*So for the One Health seminar speakers, that used to be [BUILD], but since [BUILD] has started, the One Health master's program has evolved and has been established. So we now have a One Health program which is just down the hall from us. So they've taken over those [seminars].*

The master’s program provides mutually beneficial outcomes for several partners. As a comprehensive program built into the university’s academic offerings, it led to an advanced degree for students who enroll, thereby benefiting students by making them more competitive for PhD programs. The program benefits the institution by providing both tuition funds to sustain successful STEMM training, while maintaining its innovative program components that center on supporting the needs of Indigenous and rural students. The site also mentioned their Tribal partners who saw this program as a “win–win” and agreed to donate fellowship funds to financially support students in the master’s program. Thus, reciprocity and partnership between multiple entities who perceive mutual benefits from the outcomes of innovations can motivate ways to maintain and expand initially grant-funded activities.

### Institutional resources: securing funding to continue or expand impact

A third key determinant of institutionalization is the secured institutional resources to continue grant-funded activities. Even when sites successfully achieve high compatibility and mutualism, innovations still require resources (institutional or external) to continue (Table 4). Effective strategies to obtain resources include restructuring innovations to reduce costs, finding resources that are not purely financial (e.g., physical space, staff/faculty time), utilizing resources available without institutional authorization, and obtaining more financial support both within and external to the institution.

**Table 4:** Financial resources to support continuation of BUILD Activities

Source	Examples from BUILD
Internal to institution	Endowments College Deans Provost President Vice President of Student Affairs Student Affairs Offices Academic Affairs Budget Office of Sponsored Research and Programs Office Fundraising Indirect costs from grant funding redirected to initiative
External to institution	Federal agencies: e.g., National Science Foundation (NSF), NIH Foundations State funding (separate from state appropriations to institution) Corporations/Industry: e.g., Genentech Research institution partners Sub-awardees of institutional partners awarded STEMM training grants

Sites restructured innovations to be more efficient so that they could be maintained after the grant funding period. Similar to strategies utilized in early stages of grant implementation (Cobian & Ramos, 2021), BUILD sites in the final stage of the grant continued to scale up activities to impact a greater number of participants, leverage resources to keep activities, embed activities into already existing structures on campus, and/or utilize inter-institutional partnerships. Available money, or reallocated money, was not the only type of resource that BUILD sites sought to institutionalize efforts; finding other forms of resources aside from financial support also helped. Sites were able to successfully advocate for, and obtain, shared staff time, renovate and repurpose the use of physical spaces on campus, or subsidize non-monetary institutional support with external funding.

Other sites leveraged financial budgets and resources that could be accessed without additional external support. For example, deans at Site I and Site G, who also both served as PIs on their respective BUILD grants, were able to designate spaces in their colleges to continue to provide STEMM training activities and study space for students.

Thinking creatively and tapping into other resources for financial support from both within and outside the institution was also important. For example, Site E was able to get financial support from a college dean rather than central administration. A BUILD leader there shared the following:

*It just became clear that the deans had a different kind of capacity to make things happen. Because we've been talking with [central administrators] and they were very supportive, but nothing happened... The dean really articulated clearly how this is… not just an add-on, not just something nice, but it's going to be central to the future of [the institution] to continue to track students and give them this kind of rich experience. And so that just kind of clicked with me at least that, "Oh, we've been seeking the wrong partners in this [initiative].” We need to talk to the dean. And they have the capacity, they have the budgets.*

While two deans of colleges at Site E were interested in supporting BUILD program activities, the critical factor beyond interest was their access to space and budgets, and both were able to provide a floor of office space to the program inside a building that was set to be renovated. Their team had already developed the floorplan, flow of operations for the STEMM center, and funding structure. In addition, Site E obtained state funding separate from state appropriations to the institution via a bill sponsored by a state representative. BUILD student participants testified in front of the state legislature, and, in 2021, the bill passed to fund BUILD in addition to four other educational programs in the state.

In contrast, Site A site experienced changing leadership among the president and provost, which left many BUILD leaders feeling exhausted from constantly communicating but not feeling heard or supported with resources. As one of the largest institutions in the study, BUILD participants at this site shared how the institution’s decentralized structure made it difficult to garner material resources and commitments by appealing to shared values, because every entity had differing aims. This created a political environment with few resources for which many were advocating and competing to receive. Despite this challenge, institutional agents strategically placed efforts to establish a health equity research center using BUILD funds. One participant shared:

*Well, how are we going to fund the [health equity research] Center? Some of the plans we've had, or it's taking place right now, is applying for more grants. I have a grant right now… I didn't actually write the [Center] into my grant, but now that I have it, I try to integrate the [Center] somehow just [as] an example for other researchers so they can see, "Okay, this is how the [Center] has supported this particular research project." Therefore, when you apply, if you want to, you could write BUILD as a line item there. Beyond that, they're applying for foundation grants, NIH grants, [and] grants with community partners.*

Now that the center is established, it provides opportunities to obtain grant funding and pool faculty resources together to support the center’s operations. Although Site A found an alternative path to obtaining funding, this example demonstrates that instability in institutional leadership can adversely impact efforts to institutionalize interventions. Significant and repeated changes in senior administrators can stall or prevent change agents from securing resources that are vital to sustaining a program. Budgetary challenges also created roadblocks toward the institutionalization of BUILD program elements. Site J noted budget challenges at the institutional level that required them to seek alternative sources of financial support to sustain their most effective activities.

For example, an innovative position they developed, Research Advising and Mentoring Professionals (RAMPs), landed in a precarious position despite how integral the position was to the BUILD program’s success on their campus. As a workaround, the BUILD team focused on other ways to secure RAMPs via a combination of resources:

*We've built RAMPs into other NIH proposals. There are effectively RAMP-type positions built into other proposals, non-NIH proposals, through the [state] system as a result of the [BUILD] experience. And one of the proposals, which we just got responses on recently, and we have to resubmit, but that was actually to develop a nationwide [capacity]... essentially make [our institution] the center of RAMP training. And so, it wouldn't be just institutionalizing that position. It would be trying to expand that position, initially, to where we've already tried it, primarily at tribal colleges, trying to meet that need of increasing the representation of Indigenous students.*

Here, the team used a combination of efforts to obtain grant funds, embed RAMP training to cut costs, while also hoping to create a new initiative of making the RAMPs a signature innovation where other institutions could obtain training to improve advising on their campuses. Despite institutional buy-in of the BUILD program, the financial precarity at some institutions necessitated that program leaders continue to seek external funding to sustain the program. In the absence of institutional resources, external funding will continue to be a big source of support while institutions address their financial challenges.

Finally, even with extensive political savvy and experience with creating and sustaining STEMM training activities, MSIs may still be limited by financial constraints. One participant from Site C shared the following:

*It's not that the institutional leadership doesn't support sustainability, it's just that there's no money… the leadership is very supportive of what we've been doing. But we don't have the huge endowment. It's not like they're hiding this money, and they don't want to spend it on us. There's just not the money to sustain some of this [BUILD initiative].*

Here, even with persistence from grant-funded teams, financial constraints of institutions (many of which are MSIs) ultimately influence BUILD’s institutionalization efforts.

Considering constraints, we share examples of embedded agency in practice with respect to institutionalizing BUILD activities. At the time of site visits, four sites (B, D, F, and K) successfully secured institutional funding to continue the BUILD scholar program—one of the costliest BUILD initiatives—beyond the life of the grant. The BUILD PI and team at Site D were able to secure funds to support the student scholarship program in perpetuity, in addition to institutionalizing several other activities. One team member at Site D explained that the PI was critical to obtaining commitments from student affairs and senior leadership to reallocate institutional resources:

*We have persuaded the president to use some of the designated presidential scholars to then come in and be permanently assigned for BUILD selection and BUILD type work. And that's been largely the work of [BUILD PI] lobbying with the different rounds to get those funds signed over. So the dean of students provides, and the president provides [funding for the scholarship and support program].*

If dollars were not directly available, BUILD teams found ways to successfully convince senior leaders to redirect funds from other programs or units without harming the ecosystem of the institution, and/or were able to obtain commitments for physical spaces and other forms of institutional resources such as in-kind staff re-assignments. What is additionally notable is that Site J obtained another federally funded grant to continue a similar type of scholar program, and Site I had secured some institutional funding to extend the scholar program until all current scholars had graduated. These examples demonstrate the interplay between embedded agency and resource constraints in sustaining and/or moving toward institutionalizing grant-funded initiatives.

### Sustainability as a strategy on the road toward institutionalization

Although some aspects of BUILD-developed innovations made inroads toward institutionalization, sustaining activities was more prevalent as an approach. In the final two years of grant funding, sites were already scaling down the volume of participants being served and refocusing efforts toward evaluation, dissemination, and grant writing to obtain funding necessary to sustain successful BUILD activities. A BUILD team member at Site E discussed the challenge of finding additional funds to sustain student stipends and financial support for conducting undergraduate research and attending STEMM conferences–both key components of their BUILD program:

*Everyone wants to be able to have a sunset for the funding…NIH included. But you're just passing the buck to somebody else to find that money, because the money still isn't there… if it's passed onto the institution because they know it, I mean, everyone's getting squeezed in some way… It's like the elephant in the room, essentially, everyone knows the money helps, but what do you do when you have to stop a program that has that money?*

Here, pressure to sustain grant-funded initiatives became the responsibility of the small team of faculty and staff rather than the institution or larger community of STEMM equity stakeholders. The challenge of sustaining efforts at institutions that have historically been underfunded places additional burden on the innovators of those advancing historically marginalized groups in STEMM. Extending the commitment across the institution as a requirement among funders and finding ways to relieve overburdened change agent grantees, requires working together to develop solutions to propel momentum gained from grant-funded activities that demonstrate progress.

## Discussion

Overall, this study reveals how grant-funded change agents and institutional agents leverage temporary funding from grants to create more permanent initiatives in STEMM at their institutions. This empirical focus on institutionalization (as a process and a goal) helps explain the gap between the initial infusion of funds on a college campus for sustaining innovations (Buchanan et al., 2005) and progress toward institutionalization‒ making grant-funded program activities become a vital and embedded part of the institution. This study drew upon prior frameworks of the institutionalization process in higher education (Cai, 2017; Levine, 1980; Ma & Cai, 2021) which emphasized the role of embedded agency in appealing to compatibility and mutualism. This framework theorized that the degree of compatibility (shared goals) and mutualism (satisfaction with outcomes) between the innovation and the adoptees (members of the institution) determine the degree of institutionalization. We expanded the model using evidence from the 10 postsecondary sites as they worked to institutionalize components of a comprehensive STEMM training initiative. We provided additional nuance to the originally theorized determinants of institutionalization—compatibility and mutualism (originally profitability)—by identifying strategies used by the embedded agents on campus. Some examples also reveal the challenges and struggles those institutions faced as they worked toward institutionalization.

Compatibility of a STEMM program might be perceived as too specific to an institution’s mission and norms to support as many students as possible rather than a group of students perceived to be part of niche programming. Further, norms that center teaching at such institutions might make it difficult to increase faculty research productivity. BUILD campuses created partnerships with research-intensive institutions that could sustain access for student research training, and eventually to graduate school. When institutional goals were to increase undergraduate research opportunities or work toward becoming R1, that aligned well with BUILD and contributed to institutionalization of BUILD activities. We also identified a third determinant of institutionalization‒ institutional resources, which highlights the ongoing challenge of obtaining funding and sustaining efforts (Rincon & George-Jackson, 2016). Sites employed several strategies to obtain a high degree of compatibility, mutualism, and acquisition of institutional resources, starting as early as the grant-writing stage and as late as the final years of funding to continue to work toward institutionalizing components of their respective BUILD programs.

Approaches required agency of the BUILD team and other change agents on campus. Despite constraints and the persistence of institutional norms and structures, change agents can build on their initial strengths within their local site context (Kezar & Gehrke, 2017) to shift consciousness, mobilize other actors, and engage in collective action to create change (Ma & Cai, 2021; Seo & Creed, 2002). Some sites adopted a grassroots approach to obtain buy-in from faculty and deans. Other sites had presidents, provosts, and other senior leaders involved from the beginning, or they enacted a combination of top-down and bottom-up support. Either way, institutionalization requires change agents’ action to communicate goals and successes, and to advocate for resources to maintain or expand innovations. Institutionalization requires making structural changes that last beyond a leader or specific individual change agent and may require additional support from federal, state, and private funding agencies when senior leadership at the institution is unable to provide funding, connections, or other resources to contribute to deep and pervasive institutional change.

Considering the types of institutions in this study, these findings are especially relevant to MSIs and/or postsecondary institutions that serve a diverse undergraduate student body. MSIs typically operate with limited resources, requiring administrators, staff, and faculty to be creative in utilizing and maximizing resources (Rodriguez & Calderón Galdeano, 2015). Research has often portrayed MSIs through a deficit lens by not acknowledging that these institutions operate with limited resources, pushing them to not only creatively use their existing resources but also garner external funds to create more ways of serving marginalized students (Rodriguez & Calderón Galdeano 2015). As MSIs and institutions that had less than $7.5 million of NIH funding prior to being awarded a BUILD grant, this study shows evidence of how such institutions were able to navigate contexts and institutionalize BUILD innovations despite historical underfunding.

### Lessons for grant-funded initiatives

First, appealing to compatibility at the departmental, college, or institutional level by aligning efforts with an organization’s mission, vision, values, and current institutional priorities is a key lever for obtaining institutional support to maintain programs after the grant-funding period. Program directors are faced with expectations to do “more” with fewer resources (Baber, 2015; Gomez et al., 2021). Sites that were able to utilize their agency to navigate and address compatibility and mutualism were most likely to institutionalize efforts. However, resources are still needed and may still be limited even when institutional agents outside of the grant-funded team agree on compatibility and mutual benefit of the innovation. These findings highlight the structural barriers in higher education for many MSIs, but also suggest areas to support MSIs in their efforts.

Institutional leaders outside of BUILD implementation teams (e.g., senior administrators, staff, and faculty) also play an active role, particularly in working toward compatibility and in garnering resources. There were several examples where leaders within BUILD and central campus leaders at the institution worked together. Within the structure of higher education’s opportunities and constraints, these individuals have the potential to organize and obtain or reallocate resources to maintain BUILD-led initiatives after the grant funding period.

PIs from similar sites can create communities of support and guidance as they navigate and utilize their agency to push their institutions toward transformation. A key strategy was communicating and regularly discussing initiatives with institutional leaders, especially as there is turnover in senior administration. Another key suggestion is to share innovative strategies and news regarding funding sources that are both within and external to the institutions. With respect to STEMM, there are a variety of organizations that provide different types of institutional support. For example, the American Association for the Advancement of Science (AAAS) offers the SEA Change Initiative, which aims to support voluntary transformation of colleges to foster excellent and inclusive STEMM environments (AAAS, 2026). Institutions can become members, but SEA Change also offers public access to individuals of all career stages to participate in virtual community groups for engaging in discussion and sharing resources for change.

Next, long-term vision matters. Developing and implementing an institutionalization plan can increase the likelihood of successfully institutionalizing efforts. The current study’s follow-up on the BUILD sites supports the importance of having a long-term vision for program elements that can be financially supported by the institution after a grant ends. Sustaining programs with external grant funding and donations is typical in STEMM equity efforts. However, this strategy becomes an exhausting act of going from one funding source to the next, leaving directors and STEMM program staff vulnerable to gaps in funding. A long-term plan for addressing STEMM equity may evolve and change over time but is important to provide a blueprint toward institutionalizing activities and longer-term efforts of transforming culture, daily practices and processes, and values of the institution. For example, the Diversity Program Consortium had developed Hallmarks of Success as a starting point for identifying and describing institutional aims that can be monitored over time (Gibbs et al., 2022; McCreath et al., 2017).

Last, shared leadership is critical to supporting the longevity of transformation efforts on campus. Change agents can make decisions and mobilize individuals on campus to achieve institutionalization, but the change agent requires allies, evidence of success, and administrative support. Success at the sites was more likely when several key leaders were involved. This way, even when a PI or key leader on the team left the institution or moved to another position, there was still a group that had the desire and political savvy to continue making progress. This suggests the importance of considering successor engagement and training to carry out activities.

### Recommendations for future research

Future research can further explore the process of institutionalization to better understand strategies that might be more effective than others depending on context. For example, there are only two small private institutions in this multiple case study. Private institutions and/or two-year institutions may require different strategies to institutionalize grant-funded initiatives. Additionally, future work might also examine the permanence of institutionalization by examining whether program elements that were institutionalized are either still in place a few years after the end of the grant or have transformed into new elements that still work toward addressing equity in STEMM training. Scholars who examine organizational theory and/or STEMM education have posed the question of how lasting changes must be to consider them meaningful (Lopez et al., 2022). Other research might examine institutionalization using a sociocultural lens focused on routines and behaviors (Ocasio, 2023), and how a grant-funded initiative might contribute toward an institution developing a national reputation for supporting participation in biomedical research careers. Lastly, future research can examine the interrelations between securing material resources at the institution to continue grant-funded efforts and the broader goal of institutional transformation.

## Conclusion

Leaders must manage resources and navigate politically to institutionalize change efforts on campus. Grant-funded activities can have great impact, but if a successful program does not become embedded as a vital and lasting part of the institution, then its impact is limited to the duration of the grant. This study also shows the lasting impact and provides evidence of the vital need for grant funding to catalyze an institution’s people, resources, and structures to better support biomedical career training. Institutionalizing grant-funded innovations in STEMM requires making structural changes that last beyond a grant team but might require additional support from federal agencies when senior leadership at the institution is unable to provide funding, connections, or other resources to contribute to deep and pervasive organizational change. These findings are critical now more than ever, considering evidence of the successes and challenges that institutions face, even with infusion of grant funds.

With trends showing reduced funding for STEM education projects over the past 20 years (Li et al., 2020), along with the current cuts to federal funding for comprehensive STEMM training initiatives traditionally funded by NSF and NIH, effectively “passing the buck” to other entities to pay for STEMM workforce development, efforts to streamline and expand the impact of these initiatives will be severely disrupted. The campuses discussed in the study raise expectations and can serve as a model for other institutions in their efforts to provide equal opportunity to all students they serve, including those who are first-generation college students, from low-income households, and from groups that are traditionally underrepresented in higher education. As the field shifts toward changing institutional structures and cultures rather than focusing on changing students, scholars are developing measures and methodologies that can better conceptualize institutionalization of innovations and progress toward meeting national goals to support training and talent development of the scientific workforce.

## Supplementary Information

Supplementary Material 1

Supplementary Material 2

## Data Availability

The datasets generated and/or analyzed during the current study are not publicly available due to the collaboration agreement between NIH, the Coordination and Evaluation Center (CEC), and BUILD awardees to ensure confidentiality of participants in the study. Data and materials may be available upon request to the CEC (Grant #U54GM119024) charged with keeping all data generated from the Center’s evaluation activities. An individual or group may direct their requests for data sharing to the CEC at ( [info@diversityprogramconsortium.org](mailto:info@diversityprogramconsortium.org)).

## Abbreviations

AANAPISI: Asian American Native American Pacific Islander-Serving Institution
AAAS: American Association for the Advancement of Science
ANNH: Alaska Native/Native Hawaiian-Serving Institution
BUILD: Building Infrastructure Leading to Diversity
CEC: Coordination and Evaluation Center
DPC: Diversity Program Consortium
HBCU: Historically Black Colleges and Universities
MSI: Minority-serving institution(s)
NASEM: National Academies of Sciences, Engineering, and Medicine
NIH: National Institutes of Health
NSF: National Science Foundation
PI: Principal Investigator
STEMM: Science, technology, engineering, mathematics, and medicine

## Footnotes

1 We use STEMM in this study to include medicine and medical research, and STEM to refer to work where other researchers utilize that acronym.
