# Supplementary material for: Institutionalizing grant-funded interventions: a multiple case study examining long-term investments in science, technology, engineering, mathematics, and medicine (STEMM)
Source: Int J STEM Educ. 2026 Jun 8;13(1):28. doi: 10.1186/s40594-026-00620-3 (PMC13282345; doi:10.1186/s40594-026-00620-3)
Supplement: Supplementary file 1 — Supplementary Material 1 [file 40594_2026_620_MOESM1_ESM.docx]

**Table 5**

*Interview Protocol Subset of Institutionalization Questions*

| *The NIH-funded BUILD awards differ from other NIH-funded training grants in that they aim to achieve simultaneous impact at the student, faculty, and institutional levels.*   1. From your perspective and involvement with the BUILD Initiative, what aspects of BUILD features will become a central part of what the institution does in the future and why?    1. Probes: how, and to what extent, are BUILD innovations now a *vital* part of the institution?       1. Leadership: To what extent are you involved in enhancing diversity in biomedical training as part of your strategy for driving change?       2. Infra/structure: To what extent are physical structures, financial resources, policies, and faculty reward and promotion systems in place as vested interests or commitments of the institution to promote diversity in biomedical sciences?          1. How will student interventions be maintained (student scholarships, research training, and curriculum)?          2. How will faculty interventions continue? (development and training for mentoring or teaching, research support)          3. How will institutional capacity to advance biomedical research training continue? (FTE for research courses, facilities, research support)       3. What collaborations or partnerships will be continued to achieve positive outcomes related to biomedical research for underrepresented groups? (students & faculty)          1. Funding          2. Ways that inter-institutional and intra-institutional partnerships are central to institutional mission          3. Hiring incentives/policies          4. Program elements (student/faculty) 2. What challenges continue to face the institution in terms of improving diversity in the biomedical sciences?    1. Probes: leadership challenges, financial, policies, legislative, partnership, political, etc. 3. What plans are in place to secure resources to continue to sustain BUILD innovations after the end of the grant? |
| --- |
