# Supplementary material for: Institutionalizing grant-funded interventions: a multiple case study examining long-term investments in science, technology, engineering, mathematics, and medicine (STEMM)
Source: Int J STEM Educ. 2026 Jun 8;13(1):28. doi: 10.1186/s40594-026-00620-3 (PMC13282345; doi:10.1186/s40594-026-00620-3)
Supplement: Supplementary file 2 — Supplementary Material 2 [file 40594_2026_620_MOESM2_ESM.docx]

**Table 6**

*Institutionalization Codes*

| **Code & Subcodes** | **Description/Definition** |
| --- | --- |
| **Code: Institutionalization and/or Sustainability** | *Institutionalization:* Program element(s) and/or biomedical-related efforts connected to BUILD program have become or are actively becoming part of institution permanently or for the foreseeable future (more than 5 years of commitment from institution). In other words, BUILD-related efforts are more embedded into routines, structures, and/or culture of the entire institution compared to before the BUILD program.  *Sustainability:* Sustaining BUILD program elements by other means that are not quite commitments from the larger institution, but rather departmental/faculty-led, or funded/supported by other means outside of the institution. |
| Finances | Code includes successes regarding institutionalization or sustainability related to money and financing of program elements, personnel, etc. Code can also include grant-funding to continue STEM training and/or BUILD program elements, institutional budget, state funding, fundraising via university development office, etc. |
| Research and Research Infra/structure | This code includes successes regarding institutionalization or sustainability related to institutional research productivity, including processes and infrastructure to support research. Examples include discussion of plans or actions to sustain or institutionalize research activities, an undergraduate research office, and/or investments in (lab) equipment. |
| Tenure & Promotion (policies) | This code includes successes regarding institutionalization or sustainability related to tenure and promotion. |
| Diversity Training and/or Faculty-focused Training Efforts | This code includes successes regarding institutionalization or sustainability related to diversity training and/or faculty-focused training topics (e.g., pedagogy, mentoring, DEI, anti-bias, etc.). |
| Student* Advising and/or Student* Training/Development Efforts (structures, processes, programs, workshops) | Successes regarding institutionalization or sustainability related to student advising and/or student-focused training topics (e.g., how to conduct research, career-focused workshops, etc.).  * *“Student” includes undergraduates, postbaccalaureate scholars, graduate students, postdoctoral scholars)* |
| Physical Structure(s) | This code includes successes regarding institutionalization or sustainability related to physical structures and spaces. |
| Staff/ing | This code includes successes regarding institutionalization or sustainability related to staff positions/personnel (people hired permanently to do a specific job that was part of BUILD). |
| Intra and Inter-Institution Partnerships | This code includes successes regarding institutionalization or sustainability related to current partnerships that will continue to exist. In particular, this code includes discussion of how pipeline, research, and community partnerships may continue post-grant. |
| Curriculum/courses | This code includes successes regarding institutionalization or sustainability related to academic curriculum/courses. |
| New Elements | Successes regarding institutionalization or sustainability related to passing on, transferring, scaling up, or embedding a NEW BUILD-initiated effort into other offices/programs at institution that did not exist in Phase I of grant implementation (the first five years). This can also include newly created offices/centers to pass BUILD-initiated programs to take on the work moving forward. |
| Amplifying Existing Elements | This code includes successes regarding institutionalization or sustainability related to program elements/activities that already existed pre-BUILD (BUILD helped it to grow) |
| Challenges to Institutionalization and/or Sustainability | This code includes challenges regarding the institutionalization or sustainability of BUILD program elements and/or pre-existing biomedical efforts enhanced by BUILD. |
| Lessons/Suggestions for Institutionalization or Sustainability | This code includes descriptions of lessons learned, hopes, suggestions, and recommendations (things that have not been done but desire for how things could be) when it comes to institutionalizing BUILD and/or NIH aims. This code includes use of language to express hopes or suggestions that are explicit to a BUILD-associated element. |
| **Code: Institutional Culture/Climate** | This code encompasses discussions of institutional climate as related to BUILD aims to enhance diversity for groups underrepresented in biomedical fields. This code captures attitudes toward topics such as: research, supporting marginalized populations, STEMM faculty development, etc., including how attitudes have shifted as a result of BUILD |
| Context | This code captures descriptions about the institution that participants believed were important to keep in mind to help understand STEMM training. This code also includes descriptions of the institution that were unique from other BUILD sites, and any descriptions of the BUILD team’s philosophies and approaches to grant implementation. |
| Leadership for Institutionalization &/or (top-down climate) | This code includes descriptions of how institutional leaders and acts of leadership supported/hindered institutionalization and/or sustainability of BUILD-developed activities.  This code also includes important context specific to senior administration at the institution(s) and/or leadership within the BUILD implementation team, such as turnover in roles and/or shifts in attitudes toward BUILD’s aims to sustain or institutionalize efforts. |
